# Animal Reservoirs of Zoonotic Tungiasis in Endemic Rural Villages of Uganda

**DOI:** 10.1371/journal.pntd.0004126

**Published:** 2015-10-16

**Authors:** Francis Mutebi, Jürgen Krücken, Hermann Feldmeier, Charles Waiswa, Norbert Mencke, Elizabeth Sentongo, Georg von Samson-Himmelstjerna

**Affiliations:** 1 School of Veterinary Medicine and Animal Resources, College of Veterinary Medicine, Animal Resources and Bio-security, Makerere University, Kampala, Uganda; 2 Institute for Parasitology and Tropical Veterinary Medicine, Freie Universität Berlin, Berlin, Germany; 3 Institute of Microbiology and Hygiene, Charité University Medicine, Berlin, Campus Benjamin Franklin, Berlin, Germany; 4 Bayer Animal Health, Leverkusen, Germany; 5 Department of Medical Microbiology, School of Biomedical Sciences, College of Health Sciences, Makerere University, Kampala, Uganda; University of California San Diego School of Medicine, UNITED STATES

## Abstract

**Background:**

Animal tungiasis is believed to increase the prevalence and parasite burden in humans. Animal reservoirs of *Tunga penetrans* differ among endemic areas and their role in the epidemiology of tungiasis had never been investigated in Uganda.

**Methods and Findings:**

To identify the major animal reservoirs of *Tunga penetrans* and their relative importance in the transmission of tungiasis in Uganda, a cross sectional study was conducted in animal rearing households in 10 endemic villages in Bugiri District. *T*. *penetrans* infections were detected in pigs, dogs, goats and a cat. The prevalences of households with tungiasis ranged from 0% to 71.4% (median 22.2) for animals and from 5 to 71.4% (median 27.8%) for humans. The prevalence of human tungiasis also varied among the population of the villages (median 7%, range 1.3–37.3%). Pig infections had the widest distribution (nine out of 10 villages) and highest prevalence (median 16.2%, range 0–64.1%). Pigs also had a higher number of embedded sand fleas than all other species combined (p<0.0001). Dog tungiasis occurred in five out of 10 villages with low prevalences (median of 2%, range 0–26.9%). Only two goats and a single cat had tungiasis. Prevalences of animal and human tungiasis correlated at both village (rho = 0.89, p = 0.0005) and household (rho = 0.4, p<0.0001) levels. The median number of lesions in household animals correlated with the median intensity of infection in children three to eight years of age (rho = 0.47, p<0.0001). Animal tungiasis increased the odds of occurrence of human cases in households six fold (OR = 6.1, 95% CI 3.3–11.4, p<0.0001).

**Conclusion:**

Animal and human tungiasis were closely associated and pigs were identified as the most important animal hosts of *T*. *penetrans*. Effective tungiasis control should follow One Health principles and integrate ectoparasites control in animals.

## Author Summary

Tungiasis is a neglected skin disease, frequent in resource-poor communities in the tropics. It is caused by sand fleas (also called jigger fleas) which burrow in the skin of humans and animals. Tungiasis can cause physical disabilities and the associated wounds facilitate entry of pathogens including those causing tetanus. In Brazil, presence of tungiasis in animals increased the risk and severity of the human disease. Until now, no systematic studies on animal tungiasis in Africa have been conducted. Therefore, an epidemiological study was performed in Busoga sub-region, where tungiasis in humans is very common. Tungiasis was detected in pigs, dogs, goats and a cat, respectively, in their order of significance. Animal tungiasis was strongly associated with human tungiasis and the presence of the disease in animals increased the risk of human infection by a factor of six. Our findings confirmed, for the first time, a strong correlation between the presence of tungiasis in animal reservoirs and the human population in Africa. Therefore, control of tungiasis in animals should be integrated in all interventions geared at controlling tungiasis in endemic communities.

## Introduction

Tungiasis is an ectoparasitosis that accrues from the penetration of female sand fleas into the skin. *Tunga penetrans* [1] and *Tunga trimamillata* [2] are the only species known to cause tungiasis in both humans and animals. Currently, zoonotic tungiasis is endemic in southern America, the Caribbean and sub-Saharan Africa. While *T*. *penetrans* occurs in all endemic areas, *T*. *trimamillata* has only been reported in a few countries in South America [3,4]. In the endemic areas, tungiasis in humans is heterogeneously distributed [5–7]. In Uganda, human tungiasis occurs in all regions but the prevalence appears to be particularly high in the Busoga sub-region, South Eastern, and Karamoja in North Eastern, Uganda [8]. These regions are among the poorest in the country.

Epidemiological studies carried out in resource-poor communities in Africa and South America have reported point prevalence of up to 60% among humans [6,7,9]. Hitherto, no systemic investigations have been carried out in Uganda but an impromptu outbreak investigation in some parishes of Busoga sub-region in 2010 reported prevalences of up to 73% in the general population [8]. For Karamoja, a study conducted in Napak District in different seasons, reported prevalences ranging from 18.4% at the end of the rain season to 56.6% in the dry season [10]. In poor communities, tungiasis is associated with severe morbidity [11] leading to physical disability and immobility [12]. In addition, in non-vaccinated individuals, tungiasis predisposes to tetanus and may contribute to transmission of blood borne pathogens such as Hepatitis B Virus (HBV) and HIV if non-sterile instruments are used to remove embedded sand fleas and are subsequently shared between household members [13]. Deaths from tungiasis-related complications are commonly reported in Uganda [14].

*T*. *penetrans* infects a wide range of domestic, peri-domestic and wild mammals such as pigs, dogs, cats, goats, cattle, rodents, elephants, jaguars, monkeys and even armadillos. The relevance of each of the animal species in the epidemiology of human tungiasis varies from one endemic area to another. While in urban Brazil, dogs, rodents and cats are the species most frequently infected by *T*. *penetrans*, in West Africa pigs appear to be the important animal reservoirs [3,15–17]. In Brazil, infected animals seem to increase the risk of infection in humans and are associated with a high prevalence of tungiasis at a community level [15]. Although the economic significance of *T*. *penetrans* infections in animal production has not been systematically studied, existing literature points out a significant effect on growth rate, secondary bacterial infections and defects of limbs [3]. Tungiasis may also lower product quality and hence, marketability of animals. In sows, it has been reported to cause agalactia with subsequent starvation of piglets if it affects their mammary glands [18]. Obviously, poor production and decreased marketability perpetuate community impoverishment.

To date no systematic studies have been conducted to describe the epidemiology of tungiasis in animals in East Africa. In order to identify the major animal reservoirs of *T*. *penetrans* in rural Uganda and to investigate the association between animal and human disease, a cross sectional study was carried out in ten endemic villages located in Bugiri District, Busoga sub-region. The study revealed that the prevalence of animal tungiasis was high and that the disease prevalence and parasite loads in humans and animals correlated. Pigs were identified as the most important domestic animal hosts for *T*. *penetrans*.

## Materials and Methods

### Study area, population and sampling

The study was carried out in ten villages situated in Bulidha sub-county, Bugiri district, Busoga sub-region in South Eastern Uganda. Bugiri district was purposively selected amongst the ten districts of Busoga because of the high prevalence of human tungiasis reported and confirmed during a preliminary survey. Bugiri district is about 178 km in the South Eastern direction away from the capital Kampala and lies between longitude 33°10’ and 34°00’ East and latitudes 0°6’ and 1°12’ North [19].

**Fig 1:**
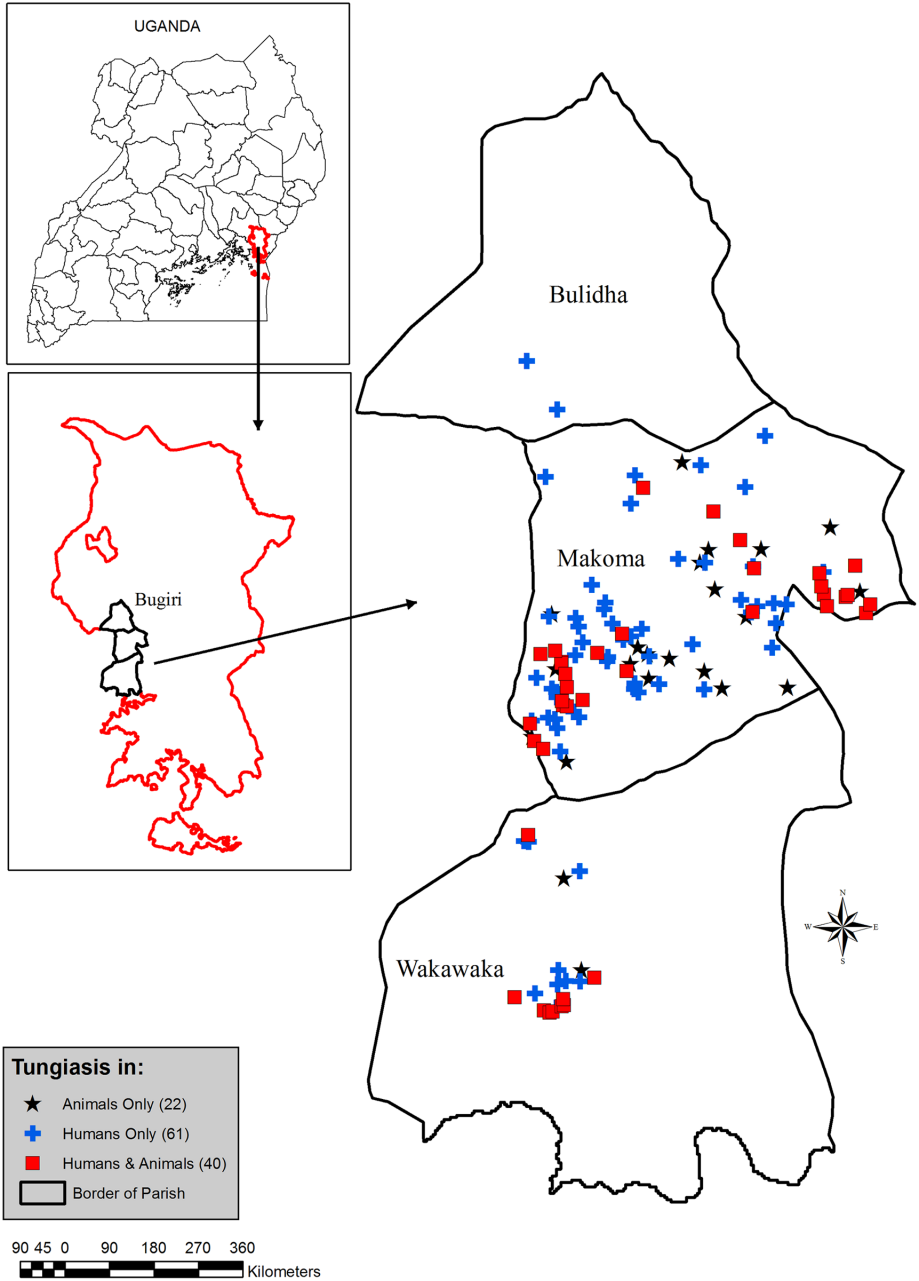
Map of study area and location of households with animal and/or human tungiasis. All study sites were located in the parishes Makoma, Wakawaka and Bulidha in the Bugiri district. Cases tended to cluster geographically.

The ten villages were; Masolya, Makoma 1, Busakira, Busano, Isakabisolo, Namungodi, Matyama and Busindha situated in Makoma parish; Kibuye and Nagongera in Wakawaka and Bulidha parishes, respectively. These were purposively selected because human tungiasis was reported to be highly prevalent by the local health personnel, a fact which was also verified during a preliminary visit. The study area and study sites with infected hosts are illustrated in Fig 1. Since there were no estimates regarding the size of the animal populations and prevalence of *T*. *penetrans* infections in the various animal species, a relatively large number of villages was included in the study. All households in the villages with at least a pig, a dog or a cat were selected for the study. All mammals accessible in the selected households were examined. Poultry were examined whenever available.

The communities were constituted by seven tribes belonging to three ethnic groups, namely Luo (Japadhola), Bantu and Nilo-hamites (Itesot). People depend on rain-fed subsistence crop and livestock agriculture. Other economic activities such as fishing in Lake Victoria and temporary work in sugar and tree plantations also contribute to households’ incomes. The major crops grown in the area were maize, cassava, rice, coffee and bananas while goats, pigs and some cattle were the major livestock reared. Dogs, cats, sheep and rabbits were other domestic animal species found in the villages. In addition, a variety of poultry especially chicken and ducks, and to some extent pigeons, turkeys as well as guinea fowls are raised. Dogs, cats and poultry roam freely on compounds throughout the year. With the exception of unweaned young animals, which roam on compounds unrestricted, other livestock are tethered on or near compounds during the crop production seasons. However, they are released intermittently after harvest with minimum food supplementation.

Homesteads are located on relatively large compounds that are close to gardens or bushes where household waste is dumped. All roads and paths are made of murram. The area experiences two rainy seasons; one between April and June and the other from August to November with an average annual rain fall of 1200 mm. Average daily temperatures range from 16.7°C to 25.1°C. Water is mainly obtained from communal boreholes, springs and shallow wells. Electricity is limited to major social facilities and less than 1% of the households have electricity.

### Study design

A cross sectional study was conducted between January 22 and March 28, 2014 which coincided with the middle and end of the dry season when the attack rate of *T*. *penetrans* usually peaks [20]. Initially, a mission was undertaken to explain the study objectives to the local medical and veterinary health personnel as well as to the local leaders. Since the study aimed to compare the significance of different animal reservoirs, only animal rearing households were included. All households with at least one pig, dog or cat were included as these species have been reported to be the most important animal hosts of *T*. *penetrans* in sub-Saharan Africa [15,17]. Households meeting the selection criteria were located with the guidance of local leaders.

In each consenting household, first a census of the animals and humans was performed. Then all mammals (dogs, pigs, cats, cattle, sheep, goats and rabbits) as well as all humans present on the compound during the investigator’s visit were examined for tungiasis. Only the poultry that were accessible were examined. If the household leader was not around, the household was visited again. Households were also revisited (up to three times) when any household member was not present; when some pigs, dogs or cats identified in the census could not be traced or when they ran away during the first investigation. In Masolya and Makoma 1, which were randomly selected among the ten study villages, all remaining households with at least one goat were also sampled to obtain an unbiased sample of goats. Wherever residents claimed to have seen rats with *T*. *penetrans*, it was attempted to trap rats in cages placed in household premises and close to the entrances of termite moulds.

### Data collection at household level

The study objectives were explained to the household heads and informed written consent was obtained. Thereafter, data was collected on social, environmental, behavioral and animal management practices through interviewing the household head and observations. Then humans and animals were examined for *T*. *penetrans*-associated lesions. Diagnosis was made clinically and humans were examined by means of a rapid assessment method [21]. To estimate the intensity of *T*. *penetrans* in humans, lesions of a randomly selected foot of up to three randomly selected children between three and eight years of age per household were counted since this is the age group with the highest intensity of infection [6,7].

To perform a thorough clinical examination, mammalian and avian species were restrained physically. However, most dogs and cats could only be examined after sedation with ketamine (Umedica Laboratories PVT. LTD, India) and xylazine (Xyla, Interchemie Werken, Netherlands). Vomiting during sedation was prevented using atropine (Gland Pharma, M. L. 103/AP/RR/97/F/R). Examination of animals was systematically performed from the head, along the trunk to the tail including the lower abdomen and the limbs through observation, hair parting and palpation. Particular emphasis was given to the paws and digits for canines and ungulates, respectively. To increase the visibility of *T*. *penetrans* lesions, the distal body parts were scrubbed with a brush and water. Sex, age and breed together with the findings of a complete clinical examination of infected animals were recorded on a standardized form. Detailed information regarding infected animals such as age was obtained by asking the owners since none of the households kept written animal records. All trapped rats were euthanized by wrapping the cages in a piece of cloth immersed in diethyl-ether.

Staging of lesions was performed according to the Fortaleza classification [22]. Viable stages were characterized by: presence of a dark brown to black spot surrounded by a reddened or swollen area (stage II) and a raised yellow to white nodule of 2–13 mm in diameter with a dark center in the skin (stage III). A brown to black, circular, raised patch in the middle of a necrotic area with or without erosions or ulcers (stage IV) and an epidermal circular shallow crater with necrotic edges (stage V) were the features considered to reflect dying or dead sand fleas [22]. In humans and animals, sores indicating that an embedded parasite had been manipulated were also documented. Photographs were taken to document the findings.

### Morphological identification of embedded sand fleas

A total of 16 (two, four and ten) embedded sand fleas were carefully extracted from some goats, dogs and pigs respectively. Animals were chosen from different villages and extraction was performed by enlarging the flea pore with tweezers. Sand fleas from humans were obtained from consenting humans who were transported by car, for treatment to Bulidha Health Center III, which was the nearest Health Unit. All extracted sand fleas were preserved in 70% ethanol and examined at the College of Veterinary Medicine, Animal Resources and Biosecurity (COVAB) using a light stereo-microscope by looking for characteristic features as described before [3].

Some alcohol-fixed sand fleas were exported to Germany, Freie Universität Berlin for scanning electron microscopy. Before electron microscopy, sand fleas were cleaned as described previously [23]. Briefly, sand fleas were dehydrated in ethanol for two hours and then kept overnight in acetone. The following day they were transferred to individual glass containers containing xylene before sonication for 30 minutes. Then, the sand fleas were washed in acetone for two hours before they were mounted on stubs, sputtered with gold and examined with the aid of a Zeiss Supra 40 VP scanning electron microscope at the Institute of Geological Sciences, Freie Universität Berlin.

### Ethical statement

Studies involving humans were conducted according to the “National Guidelines for Research involving Humans as Research Subjects” were approved by the Ministry of Health, Vector Control Division (reference no.: VCD-IRC/054). The ethical committee of the College of Veterinary Medicine, Animal Resources and Biosecurity (reference no.: VAB/REC/14/101) approved the studies involving animals. In addition, approval for both animal and human studies was obtained from the National Council of Science and Technology Uganda (reference no.: HS1621). Animal studies adhered to the “Animals (Prevention of Cruelty) act”, chapter 39, constitution of Uganda. Participation of humans was optional and written consent was obtained from household heads who also consented on behalf of their children as parents or guardians. All other adult household members (from 18 years and above) orally consented to the study, always obtained in the presence of the health workers from the nearest public health units. Only oral consent was obtained for these persons since the vast majority of participants were below 18 years and management of too many data forms was difficult under field conditions. Oral consent of the adult participants was documented on the evaluation sheets. This procedure was approved by the Ministry of Health and the National Council of Science and Technology, Uganda in the above stated documents. All humans with tungiasis were given a basic health kit consisting of a basin, a bar of soap, a sachet of detergent, individual towel, safety pins, cotton wool and an antiseptic (Dettol, Reckitt Benchiser, Dubai). Such a health kit is routinely supplied by the Ministry of Health of Uganda to affected households. Severely affected humans were transported by car to the nearest health unit for medical attention. Wounds on infected dogs, goats and pigs were cleaned with clean water and soap while antiseptic treatment was conducted using iodine tincture (SEV Pharmaceuticals Ltd, Kampala, Uganda) or a wound spray (Supona aerosol, Pfizer Laboratories (Pty) Ltd, South Africa).

### Statistical analysis

Data was entered into an Excel database (Microsoft Office, 2007) and validated by checking all entries again using the data collection tools before exportation to Stata Software package, Version 13 (Stata corporation, College Station, Texas 77845 USA) or R 3.1.2 in R Studio 0.98.1103 via a csv text file. Either Chi-square or Fisher’s exact tests were used to determine the significance of differences between proportions. In case of multiple testing, p values were corrected with the Bonferroni-Holm method as implemented in the p.adjust function of R. The Spearman’s rank correlations coefficient was calculated to establish the relationship between pairs of continuous variables. The Wilcoxon rank sum test was used to compare differences in the number of lesions between animals and/or humans groups.

Household prevalence was calculated as the proportion of households with at least one infected household member or animal to that of the total households sampled. Tungiasis prevalence among animals and humans was computed as the proportion of the number of infected animals/humans to the respective number examined. For risk factor analysis, initially, bivariate logistic regression was performed to calculate odds ratios (ORs) to assess the association between the occurrence of the infection in animals and exposure variables. Confidence intervals (95%) with no continuity correction for the prevalence [24] were computed as Wilson score intervals using www.vassarstats.net/prop1.html.

Multivariate logistic regression analyses to identify factors with effect on the chance of occurrence of tungiasis in animals in general, pigs or dogs were conducted using the”glm” function in the R software. For identification of risk factors determining the occurrence of animal tungiasis irrespective of species, the variables used included sex of household head, ethnic group of household head, education level of household head, household size, homestead size, estimated annual income, human tungiasis, manure disposal distance from compound and method of manure disposal. Others included; number of animal species in households, presence of pigs, dogs, goats, cattle, cats, chicken and other poultry respectively as well as the period of rearing animals.

For analysis of risks factors of pig tungiasis in households with pigs, the variables; “presence of tungiasis in humans”, “number of animal species”, “number of pigs”, “distance of pigs from human housing”, “presence of other ectoparasites in pigs”, “number of dogs”, “presence of tungiasis in dogs”, “number of cats”, “number of goats”, “number of cattle”, “number of chicken”, “presence of other poultry” and “sanitation of the pig dwellings (clean vs. dirty)” were initially considered. The analysis of risk factors for presence of dogs with tungiasis in households used only households with dogs and started with the variables “presence of tungiasis in humans”, “number of animal species”, “number of pigs”, “presence of tungiasis in pigs”, “number of dogs”, “presence of other ectoparasites in dogs”, “number of cats”, “number of goats”, “number of cattle”, “number of chicken” and “presence of other poultry”.

Some variable such as “presence of tungiasis in cats or goats”, “ectoparasite control in pigs”, pig management system, type of floor of pig residence or “presence of rats” were not included since the numbers of households with these states were either very low or close to 100%. Significance of individual factors was determined using the t test statistic implemented in “glm”. The Akaike information criterion (AIC) was used to compare models and the “drop1” function in R was used to progressively identify variables that could be excluded from models. Finally, pseudo-R^2^ according to McFadden was determined.

## Results

### Distribution of animal species and their management in the study villages

In the 10 villages together, 236 households were selected using the criterion of having at least one pig, dog or cat. In addition, 26 and 31 households were selected in Masolya and Makoma 1, respectively, solely on the criterion of having at least one goat. The other goat owning households had been selected due to the presence of pigs, dogs or cats. Out of 158 households with pigs in the 10 villages, three (two in Busano and one in Busindha) declined to participate. Hence, 155 pig owning households were sampled. Only one household out of 121 with at least one dog declined to participate. All the 19 households with cats in the 10 study villages were sampled. All households with at least one goat in Makoma 1 were sampled but in Masolya one household out of 48 goat owning households was excluded due to the absence of the household head during three successive visits.

Overall, there were seven species of domestic mammals (pigs, dogs, cats, goats, cattle, sheep and rabbits) and five avian species (chicken, ducks, pigeons, turkeys and guinea fowls) being reared in the area. The total number of sampled households that had the various animal species and rat trapping sites in each of the 10 villages are shown in S1 Table.

The total number of animals of the different species varied greatly in the target villages (S2 Table). Pigs (median = 40.5, range = 10–156) and goats (median = 29.5, range = 25–222) were the predominant domestic mammalian species. While chicken (median = 235, range = 140–430) and ducks (median = 22, range = 7–69) were the most abundant avian species. The median number of cattle and dogs in villages among sampled households were 12.5 (range = 3–31) and 29.5 (range = 12–53) respectively. Cats (median = 2.4, range = 0–4), sheep (median = 0, range = 0–7), rabbits (median = 0, range = 0–4), turkeys (median = 0, range = 0–10), pigeons (median = 4, range = 0–45) and guinea fowl (median = 0, range = 0–6) were rare.

There was also a considerable variation in the number of animals of each species reared per household. Pig rearing households had a median number of two pigs (range = 1–20) and those with goats had a median of 4 goats (range = 1–18). Households with dogs had a median of two dogs (range = 1–10) and those with cats had a median number of one cat (range = 1–3) per household. The highest variation occurred among chicken rearing households which had a median of eight chicken (range = 1–60). Other species were found in very few households. Although an attempt was made to trap rats from all the villages at 34 sites, only 65 rats were trapped in cages from 22 sites across five villages (S1 and S2 Tables).

With the exception of one pig rearing household (which was raising pigs intensively in a concrete floored house), the majority (154 out of 155; 99.4%) confined pigs on earthen floors during the crop growing season and released them to scavenge after harvest. Dogs, cats and chicken roamed freely with no restriction on compounds. Dogs were not housed at all and cats lived inside the human houses. Chicken and other avian species were mainly housed in earthen kitchens or inside the human house in most households (79.8%, 162 out of 203) or in provisional structures or cages on compounds (20.2%, 41out of 203). In all households goats were tethered during the day in bushes and kept at night in the kitchen, house or verandas (48.6%, n = 103); provisional structures (31.6%, n = 67) or in open spaces on pegs on the compound 19.8% (n = 42). Ectoparasite control was practiced (but with no defined regular schedule) in 12.9% (20 out of 155), 10.4% (22 out of 212) and 5% (6 out of 120) of pig, goat and dog owning households, respectively. Owners used ectoparasiticides by either washing or spraying the animals.

### Identification of neosomic sand fleas from infected animal hosts and humans

All gravid females extracted from infected animals and humans for identification exhibited a clover-like exoskeleton structure of the anterior extremity of the hypertrophic abdomen and hence were characterized as *T*. *penetrans* (S1A Fig). Scanning electron microscopy also confirmed the presence of this structure (S1B Fig)

### Animal tungiasis in the villages

Proportions of households with tungiasis in animals and humans in the study area are provided in Table 1. Animal tungiasis was detected in nine of the ten villages with an overall prevalence of 26.3% (n = 62, 95% CI 21.1–32.2%) out of the 236 households. Nagongera was the only village without any case of animal tungiasis. No animal cases were detected among the 57 additional households from Makoma 1 and Masolya which were selected on the criteria of having at least one goat to achieve a representative sample of goats in these villages. The overall prevalence as well as species-specific prevalence varied widely. The proportions of households with at least one infected animal were also highly variable among villages with a median proportion of 22.2% (range = 0–71.4%). Busindha village had the highest prevalence of tungiasis among households (71.4%, 95% CI 45.4–88.3%).

**Table 1:** Proportions of households with animal tungiasis (at least one animal) according to host species in the 10 villages.

Village	All animals		Pigs		Dogs		Goats		Cats		Humans	
	Inf. /sam.^a^	% (95% CI)	Inf. /sam.^a^	% (95% CI)	Inf. /sam.^a^	% (95% CI)	Inf. /sam.^a^	% (95% CI)	Inf. /sam.^a^	% (95% CI)	Inf. /sam.^a^	% (95% CI)
**Kibuye**	11/45	24.4 (14.2–38.7)	10/40	25 (14.2–40.1)	2/16	12.5 (3.5–36)	0/29	0	0/4	0	18/45	40 (27–54.6)
**Masolya**	18/32^b^	56.3 (39.3–71.8)	15/23	65.2 (44.9–81.2	7/20	35 (18.1–56.7)	1/47	2.1 (0.4–11.1)	0/4	0	20/32*	62.5 (45.3–77.1)
**Makoma 1**	8/30^b^	26.7 (14.2–44.5)	8/23	34.8 (18.8–55.1)	0/7	0	0/50	0	0/2	0	7/30*	23.3 (11.8–40.9)
**Busakira**	6/19	31.6 (15.4–54.0)	5/13	38.5 (17.7–64.5)	1/10	10 (1.8–40.4)	0/10	0	0/1	0	8/19	42.1 (23.2–63.7)
**Busano**	2/17	11.8 (3.3–34.3)	2/4	50 (15–85)	0/15	0	0/12	0	0/1	0	4/17	23.5 (9.6–47.3)
**Nagongera**	0/19	0 (0–16.8)	0/9	0	0/14	0	0/15	0	0/1	0	2/19	10.5 (2.9–31.4)
**Isakabisolo**	1/20	5 (0.9–23.6)	1/6	16.7 (3.0–56.4)	0/12	0	0/14	0	0	0	1/20	5 (0.9–23.6)
**Busindha**	10/14	71.4 (45.4–88.3)	9/12	75 (46.8–91.1)	3/8	37.5 (13.7–69.4)	1/10	10 (1.8–40.4)	0/4	0	10/14	71.4 (45.4–88.3)
**Namungodi**	3/25	12 (4.2–30)	3/14	21.4 (7.6–47.6)	0/13	0	0/16	0	0/1	0	8/25	32 (17.2–51.6)
**Matyama**	3/15	20 (7.1–45.2)	1/11	9.1 (1.6–37.7)	1/5	20 (3.6–62.5)	0/9	0	1/1	100 (20.7–100)	2/15	13.3 (3.7–37.9)
**Overall**	62/236	26.3 (21.1–32.2)	54/155	34.8 (27.8–42.6)	14/120	11.7 (7.1–18.6)	na^c^	na^c^	1/19	5.3 (0.9–24.6)	80/236	33.9(28.2–40.2)

^a^Infected/sampled.

^b^The numbers exclude additional households selected on the criteria of having at least one goat to avoid bias (see Materials and Methods).

^c^Not applicable.

Pigs, dogs, goats and a single cat were the only infected domestic mammalian species out of the seven examined (Table 2). Only two cases of tungiasis were detected in goats (one in Masolya and one in Busindha village). Goat tungiasis was detected in only one goat rearing household out of 97 (1%, 95% CI 0.2%-5.6%) in Masolya and Makoma 1 combined, where unbiased sampling was undertaken. The only infected cat was found in Matyama village. Rats (n = 65) and all poultry species examined were not infected even in households where other animal species and humans were heavily infected. However, many chickens were infested with the flea *Echdinophaga gallinacea*. Pig tungiasis occurred in nine of the 10 villages, while in dogs, tungiasis occurred in only five villages. In both species, the prevalence varied considerably (median = 16.2%, range = 0–64.1%; median = 2%, range = 0–26.9%, respectively). Pigs were significantly more affected than other species (pigs vs. dogs, p<0.0001; pigs vs. cats, p = 0.02; pigs vs. goats, p<0.0001. There was no significant difference in prevalence between the dogs and cats (p = 0.54), though dogs were significantly more affected than goats, p<0.0001.

**Table 2:** Prevalence of tungiasis in the four animal species which were found to be infected with *T*. *penetrans*.

Villages	Pigs		Dogs		Cats		Goats	
	Infected /Examined	% (95% CI)	Infected/ Examined	% (95% CI)	Infected/ Examined	% (95% CI)	Infected/ Examined	% (95% CI)
Kibuye	21/154	13.6 (9.1–20)	2/50	4 (1.1–13.5)	0/3	0 (0)	0/108	0 (0)
Masolya	29/63	46 (34.3–58.2)	7/43	16.3 (8.1–30)	0/4	0 (0)	1/160	0.6 (0.1–3.5)
Makoma 1	9/63	14.3 (7.7–25)	0/13	0 (0)	0/3	0 (0)	0/222	0 (0)
Busakira	10/39	25.6 (14.6–41.1)	3/26	11.5 (4–29)	0/3	0 (0)	0/25	0(0)
Busano	2/10	20 (5.7–51)	0/35	0 (0)	0/2	0 (0)	0/45	0 (0)
Nagongera	0/37	0 (0)	0/28	0 (0)	0/1	0 (0)	0/43	0 (0)
Isakabisolo	1/17	5.9 (1.1–27)	0/22	0 (0)	0/0	0 (0)	0/56	0 (0)
Busindha	41/64	64.1 (51.8–74.7)	7/26	26.9 (13.7–46.1)	0/3	0 (0)	1/55	1.8 (0.3–9.6)
Namungodi	7/39	18 (9–32.7)	0/30	0 (0)	0/1	0 (0)	0/75	0 (0)
Matyama	1/28	3.6 (0.6–17.7)	1/9	11.1 (2–43.5)	1/2	50 (9.5–90.6)	0/44	0 (0)
Overall	121/514	23.5 (20.1–27.4)	20/282	7.1 (4.6–10.7)	1/22	4.6 (0.8–21.8)	n.a.^a^	n.a.^a^

^a^For the two villages, Makoma 1 and Masolya where unbiased sampling of goats was done, the combined prevalence of goat tungiasis was 1/382 (0.26%, 95% CI 0.05–1.46%)

There was no correlation between prevalence of pig tungiasis and the size of the pig population at both household and village levels (rho = 0.09, p = 0.28 and rho = 0.3, p = 0.44 respectively). The same trend was evident for dogs (household level rho = 0.08, p = 0.4; village level rho = 0.05, p = 0.9). Among pig rearing households, there was no significant difference in the proportion of those with infected pigs between those that practiced ectoparasite control for pigs and those that did not (6 out of 20 vs. 48 out of 135, p = 0.42). This was also true for dog owning households (2 out of 6 vs. 12 out of 114, p = 0.145). The two cases of goat tungiasis occurred in households with infected pigs and humans. The only infected cat had a single lesion and was detected in a household with neither human nor other animal species tungiasis. The infected goats were three week old kids while the cat was two years old.

### Human tungiasis

Families had a median size of eight (range 1–24) members with a median of two households (range 1–8) on the same compound. Most households (89%, n = 210) had earthen floored houses which occupants occasionally smeared with cow dung to minimize dust accumulation in the houses. Of the 1766 examined humans from the 236 households, 856 (48.5%) were females while 910 (51.5%) were males. Human tungiasis was detected in all the ten villages (Tables 1 and 3). In 80 (33.9%, 95% CI 28.2–40.2%) of the 236 households (which had at least one dog, cat or a pig), at least one human was affected (Table 3). The prevalence of households with human tungiasis also varied greatly (median 27.8%, range 5–71.4%). In the additional 57 households (selected on the criterion of at least one goat after covering those selected on the criterion of possessing a dog, cat or pig) in Masolya and Makoma, 21 (36.8%) had at least one human case.

**Table 3:** Prevalence of human tungiasis in the animal rearing households.

Village	Number examined	Number affected	Prevalence (%)	95% confidence interval (%)
Kibuye	337	68	20.2	16.2–24.8
Masolya^a^	228	85	37.3	31.3–43.7
Makoma 1^a^	214	11	5.1	2.9–9.0
Busakira	164	25	15.2	10.5–21.5
Busano	144	11	7.6	4.3–13.2
Nagongera	154	2	1.3	0.36–4.6
Isakabisolo	128	2	1.6	0.43–5.5
Busindha	99	36	36.4	27.6–46.2
Namungodi	190	12	6.3	3.7–10.7
Matyama	108	2	1.9	0.51–6.5
Overall	1766	254	14.4	12.8–16.1

^a^The prevalence of human tungiasis from the additional 57 goat owning households (from Makoma 1 and Masolya) was 48/382 (12.6% CI 9.6–16.3%)

**Fig 2:**
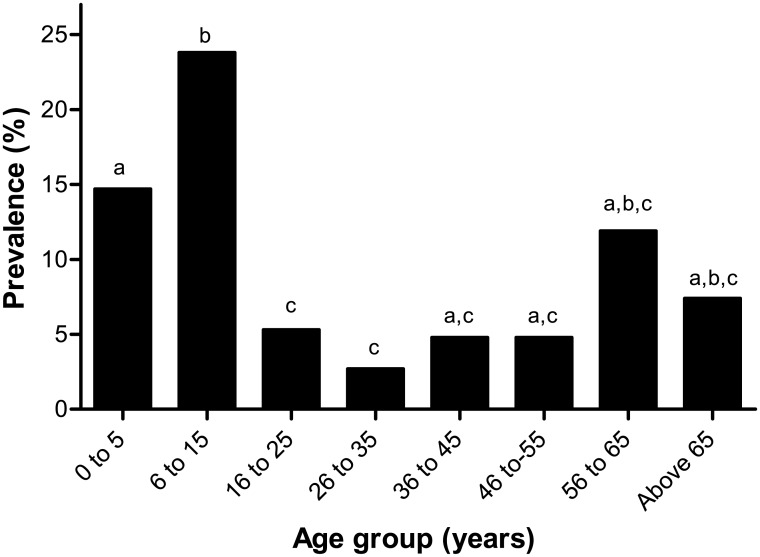
Age-specific prevalence of human tungiasis. Data with the same index letter (a, b, c) are not significantly different from each other in a Chi-square test with p values corrected for multiple testing according to the Bonferroni-Holm method.

Among humans examined for tungiasis in the 236 animal rearing households sampled, 254 (14.4%, 95% CI 12.8–16.1%) were infected but in the 57 additional households, 48 out of 382 humans (12.6%, 95% CI 9.6–16.3%) were infected (p = 0.20). Prevalences of human infections in the villages (Table 3) were significantly variable (median 7%, range 1.3–37.3%; p<0.0001). The prevalence of tungiasis was significantly higher in males (n = 154, 16.9%, 95% CI 14.6–19.5%) than females (n = 100, 11.7%, 95% CI 9.7–14.0%; p = 0.001). Children (0–15 years) had a significantly higher prevalence of tungiasis than other humans above 15 years (20.3% vs. 5.5%; p<0.0001). The prevalence of human tungiasis was highest in children of 6–15 years (Fig 2). Overall, the variations in prevalence of tungiasis among age groups were statistically significant (p<0.0001). There was also a significantly higher prevalence in the two youngest age groups compared with the middle age groups and a clear peak in the age group of 6–15 years (Fig 2).

### Relationships in occurrence of tungiasis between animals and humans

In 40 (17%, 95% CI 12.7–22.3) of the sampled households, animal and human tungiasis coexisted. These constituted 50% and 64.5% of the households with human and animal tungiasis respectively. Animal tungiasis increased the odds of the occurrence of human tungiasis in households by six times (OR = 6.1, 95% CI 3.3–11.4%; p<0.001) and vice versa. In Busindha, all animal rearing households that had infected animals also had infected humans. In Isakabisolo, the proportions of households with infected animals were similar to that of humans but infections were detected in different households, i.e. there were households where only human or only animal tungiasis was detected. In other villages the proportions differed (Table 1).

**Fig 3:**
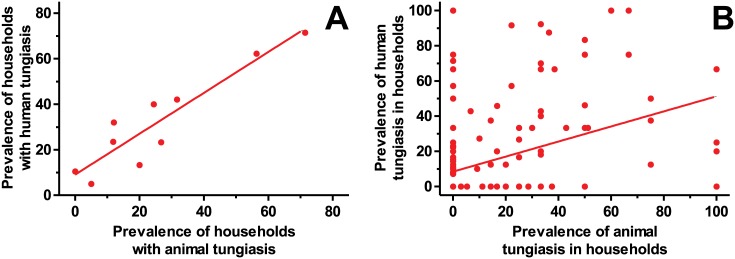
Correlation of prevalence of animal and human tungiasis. (A) Prevalence data of tungiasis in households was analyzed on the village level (rho = 0.85, p = 0.002). (B) Prevalences of tungiasis in humans and animals were compared on the household level (rho = 0.44, p<0.0001).

Overall the proportions of households with human tungiasis did not differ significantly from those with animal tungiasis in the 10 villages (p = 0.07). A strong correlation existed between the prevalence of households with human tungiasis and those with animal cases within the 10 villages as illustrated in Fig 3A. (rho = 0.85, p = 0.002). At household level, the prevalence of animal tungiasis correlated with human tungiasis prevalence (rho = 0.4, p<0.001) as shown in Fig 3B. Also, at household level, the prevalence of tungiasis in the mostly affected animal species strongly correlated with human prevalence (dogs rho = 0.34, p = 0.0002; pigs rho = 0.5, p<0.001).

### Intensity of infection in animals

**Fig 4:**
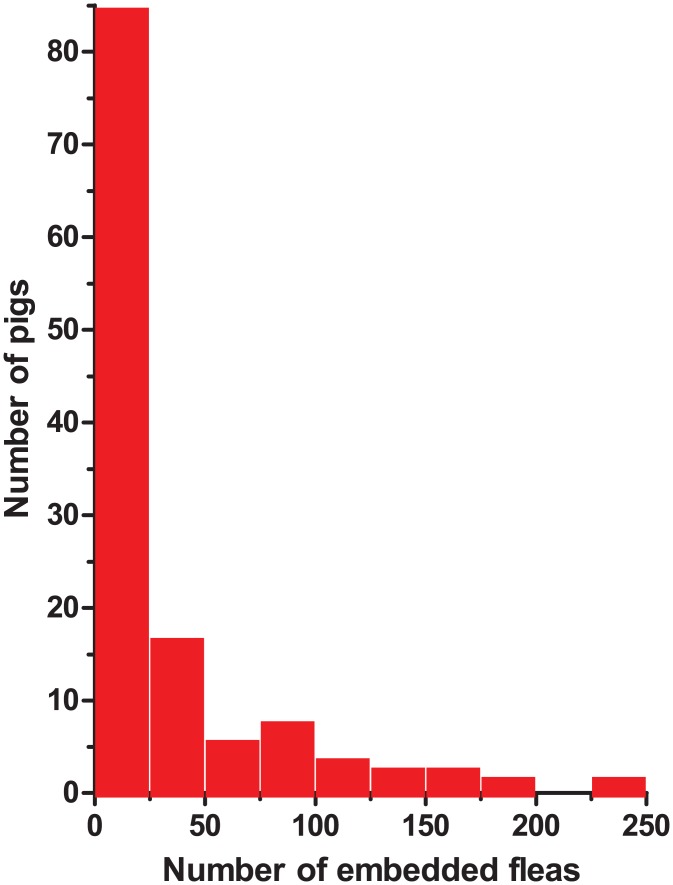
Distribution of the number of embedded sand fleas in pigs; pigs with tungiasis of all villages combined.

Pigs had the highest parasite load followed by goats and dogs: median = 8 lesions (inter-quartile range (IQR) = 3–30; range of 1–246 lesions per pig); goats median = 20 (6 and 34 lesions in 2 goats); dogs median = 2 (inter quartile range = 2–3; range = 1–8). The only affected cat had a single non-viable lesion. Of the 3357 lesions in pigs, 2243 (66.8%) were viable (Fortaleza stage IIa-IIIb) and 1114 (33.3%) were non-viable (Fortaleza stage IV). The number of lesions per pig was highly variable (Fig 4). Among the infected pigs, those which had the highest infection intensity (>30 lesions, n = 30, 24.8%) presented 79% of the total number of embedded sand fleas.

**Fig 5:**
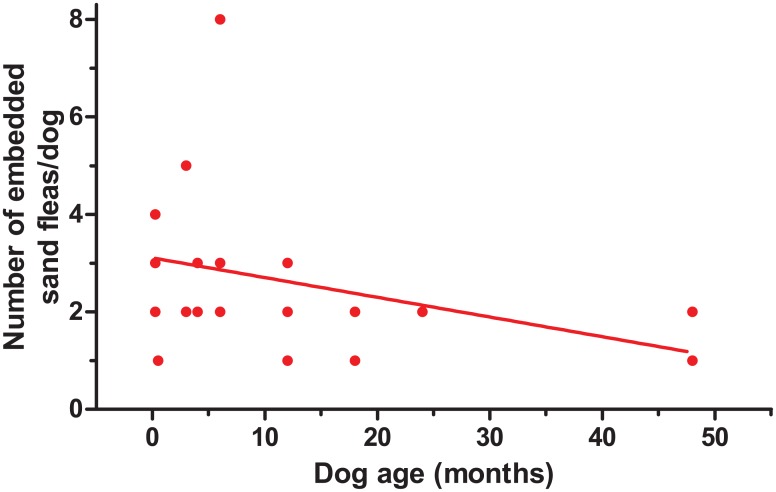
Correlation between age of dogs infected with *T*. *penetrans* and the number of embedded sand fleas (rho = -0.47, p = 0.039).

The 20 infected dogs had a total of 53 lesions of which 32 (60.4%) were viable while 21 (39.6%) were non-viable. Occasionally, it was observed that dogs bite at flea lesions and exteriorized the fleas with their teeth. Out of the 20 dogs only 2 (10%) had ≥5 lesions while the other 18 had light infections (1–4 lesions per dog). Of the total 40 lesions found on the two goat kids, 27 (67.5%) were viable while the other 13 (32.5%) were non-viable. In pigs, no correlation was observed between age and the total number of lesions (rho = 0.014, p = 0.88) but in dogs the number of lesions per dog significantly decreased with age as shown in Fig 5. (rho = -0.47, p = 0.039). The number of lesions did not differ between sexes: female median 10 (IQR 3–39) vs. male median 6 (IQR 3–30) in pigs (p = 0.37); female median 2 (IQR 2–3) vs. male median 2 (IQR 2–3) in dogs (p = 0.88).

### Intensity of infection in children three to eight years of age

In the 236 households, sand flea lesions were counted in 111 infected children aged three to eight years, the age group known to have the highest intensity of infection. These included 62 boys and 49 girls. A total of 340 lesions were documented from one randomly selected foot of these children. The median number of lesions per infected child was 2 (range 1–18). The number of lesions per foot never differed significantly between boys (median 2, range 1–18) and girls (median 2, range 1–8; p = 0.42).

### Comparison and correlations of parasite loads between species

**Fig 6:**
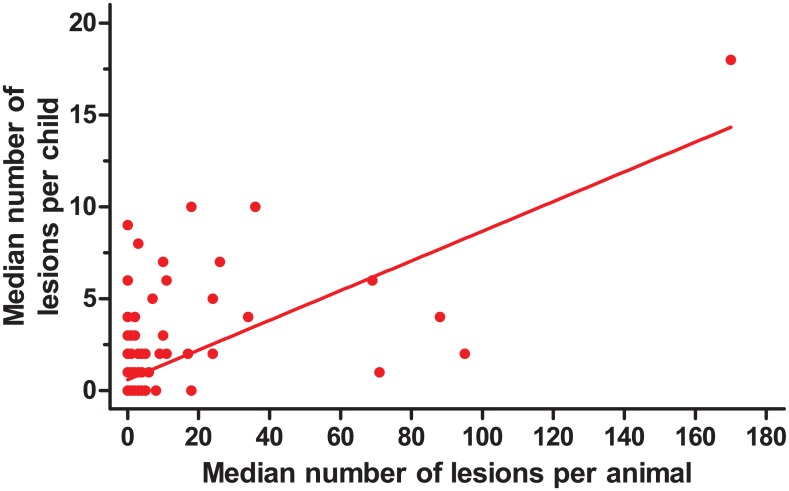
Correlation between the median number of lesions in infected children and infected household animals. The median number of lesion in children 3–8 years old on one randomly selected foot was plotted against the median number of lesions in animals from the same household (rho = 0.47, p<0.001).

Pigs had a significantly higher number of lesions than other species combined (median 8, range 1–246 vs. median 2, range 1–34; p = 0.0002). Accordingly, the number of lesions was also significantly higher for pigs than dogs (p < 0.0001). The median number of lesions in infected animal species strongly correlated with the median number of lesions in children three to eight years of age at household level as illustrated in Fig 6 (rho = 0.47, p<0.0001). The prevalence of human tungiasis at household and village levels correlated strongly with the number of lesions in pigs at the respective levels (rho = 0.5, p<0.0001; rho = 0.8, p = 0.002). At household level, an increase in human tungiasis intensities corresponded with an increase in the odds of occurrence of animal infections (OR = 1.8 CI 1.4–2.2, p<0.0001) and vice versa (OR = 1.3 CI 1.1–1.4, p < 0.0001).

### Effects of management practices on animal tungiasis

Of the infected 121 pigs, only 18 (15%) had ever received ectoparasite treatment and none of these had a well-defined interval of treatment. The period between the last time of pig treatment and the examination date ranged from one week to 10 weeks. Nine pigs had received ectoparasitic treatment between one and two weeks before the examination date while the rest (9 pigs) had received treatment between five and 10 weeks prior to the examination date. Ectoparasiticides which had been used on infected pigs included pyrethroids (6), amitraz (6) and a traditional concoction of molasses and a local gin (*waragi*) which was used on one pig. Three pig owners could not recall the type of ectoparasiticide they had used to treat five of the infected pigs.

Although, the total number of sand flea lesions per infected pig was lower in pigs treated with ectoparasiticides than the untreated (treated median 6.5, IQR = 3–13 vs. untreated median 8, IQR = 3–39), the difference was not significant (p = 0.34). For treated pigs, there was no correlation between the number of embedded sand fleas and the time span since when the pigs received the last ectoparasiticide treatment (rho = -0.16, p = 0.53).

Pig dwellings were located at a median distance of 11 meters (range 0–50 m) from the edge of human compounds. The distance of pig dwellings (places of pig confinement) from human compounds had a weak positive correlation with the total number of lesions per pig (rho = 0.18, p = 0.043).

Ectoparasite control had been attempted for only two of the infected dogs (10%) with no definite control interval. While one dog had received the ectoparasiticidal treatment one week before the examination date, the other had received the same treatment four weeks ago and in all cases α-cypermethrin 10% was used. Incidentally, the more recently treated dog had more lesions than the dog treated three weeks before (three lesions vs. one lesion). Neither the two infected goat kids nor the infected cat had ever received any ectoparasiticidal treatment.

### Risk factors for animal infections in households

A bivariate analysis of risk factors was undertaken (S3, S4 and S5 Tables). Factors strongly associated with occurrence of tungiasis in all animals irrespective of species; pigs and dogs within households are summarized in Table 4. Occurrence of infected animals in households was strongly associated with human infections (OR = 6.0, 95% CI 2.4–9.1; p < 0.0001) and presence of pigs in a household (OR = 5.8, 95% CI 2.5–13.5; p < 0.0001) among other factors. For households with infected humans, the risk of animal tungiasis increased with the number of infected humans. One to four infected humans in households compared to none increased the risk by five times (OR = 5.0, 95% CI 2.6–9.5; p < 0.0001) but presence of 5–12 infected individuals raised the odds to 12 times (OR = 12.2, 95% CI 4.1–35.8; p < 0.0001). Pig tungiasis occurred in strong association with dog tungiasis (OR = 7.4, 95% CI 1.5–36.9; p = 0.02) and human tungiasis (OR = 7.0, 95% CI 3.4–14.7; p<0.0001) while presence of infections in dogs was also strongly influenced by human tungiasis (OR = 12.2, 95% CI 2.6–57.4; p = 0.002) and pig infections (OR = 5.6, 95% CI 1.7–18.2; p = 0.004).

**Table 4:** Risk factors of tungiasis in animals at household level.

Animal tungiasis risk factors at household level		
Factor	Odds ratio (95% CI)	p-value
Human infection	6.1 (3.3–11.4)	0.0001
Presence of pigs in a household	5.8 (2.5–13.5)	0.0001
Young household heads (15–35 years)	2.1 (1.0–4.4)	0.05
Many animal species (5–9)	2.2 (1.0–5.0)	0.05
Medium household size (6–10 people)	3.0 (1.2–7.5)	0.02
Large household size (>10 people)	3.0 (1.1–8.6)	0.04
Occurrence of human disease in three months	4.6 (2.4–9.1)	0.0001
**Pig tungiasis risk factors**		
Human tungiasis	7.0 (3.4–14.7)	0.001
Medium herd size (4–6 pigs)	3.3 (1.3–8.1)	0.01
Presence of other ectoparasites	10 (1.3–77.4)	0.03
Presence of dogs	2.1 (1.1–4.3)	0.03
Manure soiled pig dwellings	2.9 (1.0–8.1)	0.04
Dog tungasis	7.4 (1.5–36.9)	0.02
**Dog tungiasis risk factors**		
Human tungiasis	12.2 (2.6–57.4)	0.002
Presence of cats	4.6 (1.0–20.8)	0.05
Pig tungiasis	5.6 (1.7–18.2)	0.004

**Fig 7:**
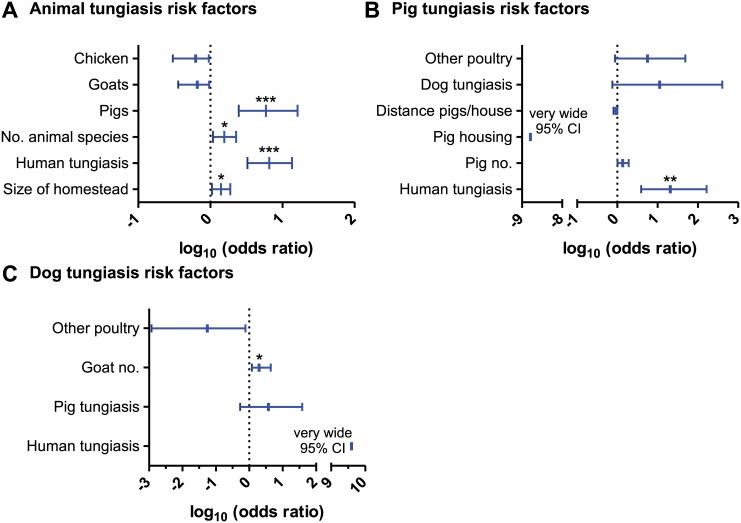
Multivariate risk factor analysis for animal tungiasis. Variables were analyzed by logistic regression and were eliminated stepwise to obtain an optimized model in terms of a minimal AIC value. Data are presented as odds ratios with 95% confidence intervals. (A), (B) and (C) show risks factor analysis for the presence of tungiasis in a household in any animal species, pigs and dogs, respectively. *** p < 0.001; **, p < 0.01; *, p< 0.05. When confidence intervals were very wide, they were not plotted.

In multivariate analysis, presence of animal tungiasis in households was strongly influenced by presence of human tungiasis (OR = 6.5, p < 0.0001) and presence of pigs (OR = 5.9, p = 0.0002) as illustrated in Fig 7A. In addition, the number of animal species reared in the household (OR = 1.6, p = 0.02) and the size of the homestead (OR = 1.4, p = 0.02) significantly increased the odds detecting animal tungiasis among households. Slightly but non-significant protective effect was observed in association with the presence of chicken and goats in the households. However, the overall model had a poor to moderate fit (McFadden pseudo R^2^ = 0.25).

Due to the overall low fit of the animal tungiasis model, separate models were estimated for pig and dog tungiasis. The odds of households to have pigs infected with *T*. *penetrans* were significantly increased if there was also human tungiasis as illustrated in Fig 7B (OR = 2.1, p = 0.001). The final model showed an excellent fit (McFadden pseudo R^2^ = 0.82). It also included the variables; “presence of other poultry”, “dog tungiasis” and “pig herd size” which all increased the odds of pig tungiasis although their effects were all not significant. In addition, housing of pigs had a very low OR of 1.6×10^−9^ suggesting that it has strong protective effect against pig tungiasis. However, this effect was not significant due to the very low number of households with housing for pigs hence a very wide 95% CI.

The same analysis for the presence of dog tungiasis in households (Fig 7C) suffered from the low number of households with dog tungiasis identified in the study (n = 14). Nevertheless, the overall fit of the model was very good (McFadden pseudo R^2^ = 0.71). The variable with the strongest influence on the odds of households to have dog tungiasis (human tungiasis, OR = 4.0 × 10^9^ had a very wide 95% CI. The number of goats significantly increased the odds (OR = 2.0, p = 0.04) while pig tungiasis had a non-significant influence. Presence of poultry other than chicken appeared to be slightly protective but this effect was also not significant.

## Discussion

Various domestic, peri-domestic and sylvatic mammals have been reported as suitable hosts of zoonotic sand fleas. Domestic and peri-domestic animals which are reportedly more important in the epidemiology of tungiasis than sylvatic reservoirs include cats, dogs [16], rats, mice [15,17], pigs [17,25,26], goats [27], sheep and cattle [3]. The significance of the different animal reservoirs for *T*. *penetrans* differs between endemic areas. While cats, dogs and peri-domestic rodents have been reported to be the most important animal hosts for *T*. *penetrans* in Northeast Brazil [15,16,25], pigs have been identified as the major reservoirs in a single study in West Africa [17].

Studies looking for an association between animal and human tungiasis are scarce. Previous studies performed in Northeast Brazil [15,16,25] and Nigeria [17] were limited to one or two small communities, covered a few animal species and were less systematically conducted compared to this study. For the first time, it was attempted to identify the risk factors that determine the occurrence of animal tungiasis. Since the study area is typical of many rural settings in Uganda, the results can presumably be extrapolated to other rural communities in the country and probably East African countries.

Pigs were the animal species predominantly affected and they also had the highest intensity of infection. These findings corroborate observations made in Nigeria [17]. Since both, the prevalence of tungiasis in pigs at village and household level as well as the intensity of infection correlated strongly with the respective measurements in humans, it can be proposed that pigs are the reservoir hosts that contribute most to the occurrence of tungiasis in humans in the study area. Dogs, especially puppies, cats and goats had very low prevalences of tungiasis. Hence they are alternative hosts of *T*. *penetrans* in the study area. To what extent these species contribute to amplification and propagation of *T*. *penetrans* in humans is unknown.

Cattle and other mammalian species with the exception of pigs, dogs, cats and goats were not found to carry *T*. *penetrans*. However, since only a few of them were sampled, it cannot be excluded that they also act as reservoirs of *T*. *penetrans* in the study area and other areas of Uganda. Reports from South America indicate that cattle seem to be particularly susceptible to *T*. *trimamillata* infection and *T*. *penetrans* infection is encountered usually as a co-infection [3]. In the study area, the absence of tungiasis among cattle could also be attributable to the higher rate of ectoparasite control in cattle (46% of the cattle owning households) than for other species.

Chicken and other avian species have been suggested as hosts for tungiasis [25]. However, whether chicken and other avian species are actually appropriate *T*. *penetrans* hosts remains to be demonstrated. In this study, many chicken examined had *E*. *gallinacea* infestations which people mistook for *T*. *penetrans*. Since chicken stay close to human dwellings (in the study area all chicken are strayed on the compound and the majority spent nights either in human houses or in small separate kitchen buildings), one would expect that if chicken were susceptible, they would have the highest burden of *T*. *penetrans* infections in infected households compared to other susceptible species. It seems that thick feathers on the chicken body and the scaly legs are barriers to sand flea penetration.

The current study did not detect any case of tungiasis among the 65 rats trapped from five villages, a finding which contrasts with reports from Brazil and Nigeria where rodents were identified as important reservoirs of *T*. *penetrans* [15,17]. In marked contrast to this observation, 28% (n = 82) of the household heads interviewed claimed to have seen rats with tungiasis either in their houses or homesteads. Since the residents’ abilities to discern tungiasis from other parasitic diseases of rodent feet, such as other flea species or even myiasis, may be low, their assertions have to be considered with great caution and further studies are recommended.

Both cases of tungiasis in three week old goat kids occurred in association with heavy human and pig infections in the respective households. In contrast, adult goats were not infected even in premises with many infected humans and pigs with high intensity of *T*. *penetrans*. Although goats were the most abundant domestic mammalian species in the area, they appear to be of no epidemiological significance in the transmission of *T*. *penetrans*. A soft hoof wall and the skin around the coronary band of the digits together with the practice of leaving kids to roam freely on compounds coupled with sheltering kids in human or close to human dwellings could explain the infections in kids. In contrast, adults have hard hooves and are tethered in bushes most of the time during the day.

Tungiasis in goats has been reported in a few countries in South America. As is the case with cattle, it was mostly due to *T*. *trimamillata* with some reports of co-infections with *T*. *penetrans* [3]. A study on animal reservoirs of *T*. *penetrans* in rural communities in Ethiopia reported a prevalence of 3.2% in goats in one out of four study communities. In contrast, tungiasis was detected in sheep in all communities with a prevalence of up to 29.5% [28]. The few sheep encountered and examined in this study were not infected. It remains unclear why prevalences greatly differed between the two small ruminant species in Ethiopia despite their anatomical similarities.

All households involved in the study had outside resting places for residents, either under a tree or below an erected temporary shade. Since pigs are kept away from human houses most months of the year, the high prevalence of tungiasis in pigs strongly suggests that transmission of *T*. *penetrans* also occurs distant to the compounds. Pigs are mostly reared on earthen floors with minimal environmental sanitation management. For this reason, dirty pig dwellings were identified as a risk factor for pig tungiasis at least in the bivariate analysis. The organic material may favor the off-host development of *T*. *penetrans* thus predisposing pigs to heavy infections [20]. This was in fact indicated by the high proportion of viable lesions particularly in pigs as compared to dogs. Of course, the practice of allowing them to roam on human compounds also increases the risk of their infection and also facilitates the shedding of sand flea eggs in human dwellings when pigs are infected.

The prevalence of tungiasis and the infection intensity among pigs reported in this study in some villages were comparable to those reported in Nigeria [17] but were much higher than those in Brazil [25]. Pigs may be very susceptible to sand flea infections because of the highly vascularized coronary band and large bulb at the sole of the hoof with a soft skin cushion [3]. Unlike dogs, which are never confined, pig movements are restricted during the crop growing rainy season and intermittently during the dry season. Hence, minimal movement-related thickening of the bulb epidermis takes place. This may explain the lack of variation in the infection intensities between piglets and adult pigs.

Although the prevalence of tungiasis in dogs was the second highest after that of pigs, dogs were considerably less frequently infected than in previous studies in Nigeria and Brazil [15–17]. Therefore, dogs appear to be less important as animal reservoirs in Uganda than in Nigeria and Brazil. Moreover, the number of dogs in Ugandan rural villages is comparatively small. The significance of dogs in the epidemiology of *T*. *penetrans* infections in the Ugandan setting generally decreased with dog age probably because of the increasing thickness of the keratin layer of the foot pads as the dogs mature. Since dogs were not restrained, they moved a lot between households and even between villages. This would incite hyperkeratosis of the foot pads. Additionally, mature dogs tend to bite and exteriorize the embedded sand fleas. Despite the low prevalence of dog tungiasis and decreasing infection intensity in older dogs, dogs might be epidemiologically important because they might distribute flea eggs in a much wider range than pigs. Detection of tungiasis in a single cat underlines previous observations that cats may act as reservoirs of *T*. *penetrans* [15]. However, the number of cats is low in most rural communities of Uganda.

The patterns of human and animal infections were similar in the study area among animal rearing households i.e. the prevalence and infection intensity strongly correlated. This, together with their geographical coexistence (Fig 1), suggests an inter-linkage between animal and human *T*. *penetrans* infections in Uganda. This however requires validation by systematically sampling humans. Since the focus of the present study was on identification of important reservoir hosts of zoonotic tungiasis, only animal rearing households were included. Also co-existence of infections in both humans and animals may indicate a common source of infection which was not directly examined in this study. However, occurrence of tungiasis in animals increases environmental contamination which in turn may increase the prevalence and intensity of infections in humans and vice-versa resulting in the strong correlation. The sharing of *T*. *penetrans* infections between humans and animals is most likely facilitated by the practice of allowing animals to roam freely with minimal confinement.

Households with pigs, cats and dogs had higher odds of having at least one infected animal, a finding corroborating earlier studies which identified these animal species as risk factors for human infections [15–17]. Animals from households with young household heads (15–35 years) were more at risk than those from households with older heads in the bivariate analysis. This relationship may be indirectly related to the presence of children, who are particularly at high risk in such households; lack of adequate knowledge to control sand fleas and probably lack of adequate financial resources to ensure adequate hygiene and environmental sanitation. The strong association of animal infections with large household sizes is probably also indirect and may be due to the fact that such households have many children below 15 years who constitute the most vulnerable group of humans to *T*. *penetrans*.

The presence of many animal species in households may contribute to poor environmental sanitation and overall poor animal management particularly regarding ectoparasite control. Poor environmental sanitation confers favorable conditions for off-host sand flea development and propagation [29]. The reasons for the association between *T*. *penetrans* and other pig ectoparasites such as lice in this study are not known as the ecological interactions between these parasites have not been studied yet. However, the factors that favor the occurrence of ectoparasites in general such as poor environmental sanitation and lack of ectoparasite control probably contribute to high prevalences of both; sand fleas and other ectoparasites.

In the multi-variate analyses, only a few variables remained statistically significant suggesting that the interaction of many factors predispose to animal on the household level. Nevertheless, multivariate logistic regression also revealed a very strong association of human and animal tungiasis in households and in particular the close connection between human and pig tungiasis. The role of dogs in the epidemiology of *T*. *penetrans* in Uganda could not be unequivocally described. On the one hand, the OR associated with dog tungiasis were always very high but on the other hand, effects were not significant due to the low number of infected dogs. Larger data sets, probably involving several transmission seasons, would be required to statistically confirm the role of dogs in the epidemiology of animal and human tungiasis.

The study was cross sectional in nature; hence seasonal patterns could not be demonstrated. Also the study only established the human tungiasis burden in animal rearing households with the most important animal hosts of *T*. *penetrans*. The findings therefore may not reflect the situation in the general population. Additionally, it was only possible to demonstrate the association between human and animal tungiasis but causal relationships are difficult to determine. While pigs could be confirmed as epidemiologically important reservoirs, the role of other animal species remained unresolved either due to low number of cases (dogs, cats) or because they were not systematically sampled (e.g. cattle).

### Conclusions

This study demonstrated a strong correlation between animal and human tungiasis in animal rearing households, which both occurred with high prevalence in rural endemic villages of Uganda. Pigs were identified as the major hosts of *T*. *penetrans*. An effective tungiasis control strategy; therefore, calls for an integrated One Health approach. In addition to treatment of humans and environmental sanitation, ectoparasite control should be encouraged among animal owners to eliminate animal infections. However, there is still need to evaluate the therapeutic and prophylactic effects of commercial pesticides against *T*. *penetrans*

## Supporting Information

S1 Fig Hypertrophied sand flea from a pig (stage III of Fortaleza Classification). (A) Light microscopic picture showing the clover leaf-like moulding of the anterior extremity of the first hypertrophic segment which is a characteristic feature of *T*. *penetrans* (Mg x 10) [3,22]. All sand fleas sampled showed this structure. (B) The same structure is also visible using scanning electron microscopy.
(PDF)

S1 Table Number of households in which the various animal species and humans were examined. (PDF)

S2 Table Number of animals and humans sampled in the 10 villages. (PDF)

S3 Table Animal tungiasis risk factors at household level. This table illustrates the detailed bivariate analysis of factors of tungiasis in animals at household level (herds).
(PDF)

S4 Table Pig tungiasis herd risk factor analysis. (PDF)

S5 Table Dog tungiasis household risk factor analysis. (PDF)
