# Supplementary material for: Animal Reservoirs of Zoonotic Tungiasis in Endemic Rural Villages of Uganda
Source: PLoS Negl Trop Dis. 2015 Oct 16;9(10):e0004126. doi: 10.1371/journal.pntd.0004126 (PMC4608570; doi:10.1371/journal.pntd.0004126)
Supplement: S1 Fig — (A) Light microscopic picture showing the clover leaf-like moulding of the anterior extremity of the first hypertrophic segment which is a characteristic feature of T. penetrans (Mg x 10) [3,22]. All sand fleas sampled showed this structure. (B) The same structure is also visible using scanning electron microscopy. (PDF) [file pntd.0004126.s001.pdf]

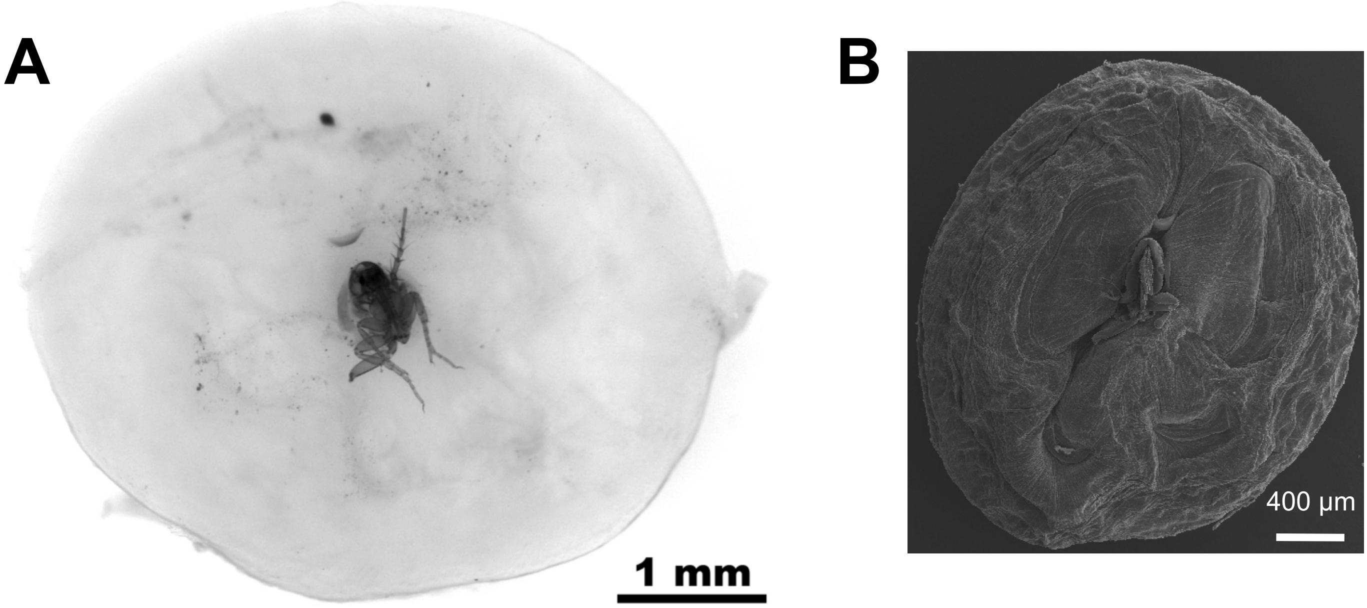

S1 Fig. Hypertrophied sand flea from a pig (stage III of Fortaleza Classification). (A) Light microscopic picture showing the clover leaf-like moulding of the anterior extremity of the first hypertrophic segment which is a characteristic feature of *T. penetrans* (Mg x 10) [3,22]. All sand fleas sampled showed this structure. (B) The same structure is also visible using scanning electron microscopy.
