# Supplementary material for: Animal Reservoirs of Zoonotic Tungiasis in Endemic Rural Villages of Uganda
Source: PLoS Negl Trop Dis. 2015 Oct 16;9(10):e0004126. doi: 10.1371/journal.pntd.0004126 (PMC4608570; doi:10.1371/journal.pntd.0004126)
Supplement: S1 Table — (PDF) [file pntd.0004126.s002.pdf]

S1 Table: Number of households in which the various animal species and humans were examined[a]

Species in households	Villages										Total	(%)
	Kibuye	Masolya	Makoma 1	Busakira	Busano	Nagongera	Isakabisolo	Busindha	Namungodi	Matyama		
**Pigs (included/sampled)**	40/40	23/23	23/23	13/13	6/4	9/9	6/6	13/12	14/14	11/11	157/155	98.7
**Dogs (included/sampled)**	16/16	20/20	7/7	10/10	15/15	14/14	12/12	8/8	13/13	6/5	121/120	99.2
**Cats (included/sampled)**	4/4	4/4	2/2	1/1	1/1	1/1	0/0	4/4	1/1	1/1	19/19	100
**Goats (included/sampled)**	29/29	48/47[b]	50/50[c]	10/10	12/12	15/15	14/14	10/10	16/16	9/9	213/212	99.5
**Cattle (included/sampled)**	6/6	9/9	4/4	2/2	3/3	7/7	5/5	5/5	1/1	5/5	47/47	100
**Sheep (included/sampled)**	0/0	1/1	1/1	0/0	0/0	0/0	0/0	0/0	0/0	0/0	2/2	100
**Rabbits (included/sampled)**	2/2	0/0	1/1	0/0	0/0	0/0	0/0	0/0	0/0	0/0	3/3	100
**Chicken (included/sampled)**	36/25	28/21	26/21	14/14	15/14	16/15	20/18	14/14	20/17	14/13	203/172	84.7
**Ducks (included/sampled)**	14/13	2/2	6/5	5/4	3/3	8/8	6/5	2/2	3/3	2/2	51/47	92.2
**Turkeys (included/sampled)**	1/1	0/0	1/1	0/0	0/0	0/0	0/0	1/1	0/0	0/0	3/3	100
**Pigeons (included/sampled)**	3/2	2/1	4/4	0/0	0/0	2/2	1/1	1/0	3/3	1/0	17/13	76.5
**Guinea fowls (included/sampled)**	0/0	0/0	0/0	0/0	0/0	0/0	0/0	1/1	2/2	2/0	5/3	60
**Rats[d]**	3	5	7	5	2	2	1	5	2	2	34	-
**Humans (included/sampled)**	45/45	32/32	30/30	19/19	19/17	19/19	20/20	15/14	25/25	16/15	239/236[e]	98.8

[a]The presence of animal species among households was not mutually exclusive
[b]Number includes additional 26 households selected on the criterion of having at least one goat
[c]Includes additional 31 households selected on the criterion of having at least one goat
[d]Number of sites where rat traps were positioned for at least three days per site
[e]Excludes additional households sampled in Makoma 1 and Masolya; see methods
