# Supplementary material for: Animal Reservoirs of Zoonotic Tungiasis in Endemic Rural Villages of Uganda
Source: PLoS Negl Trop Dis. 2015 Oct 16;9(10):e0004126. doi: 10.1371/journal.pntd.0004126 (PMC4608570; doi:10.1371/journal.pntd.0004126)
Supplement: S2 Table — (PDF) [file pntd.0004126.s003.pdf]

S2 Table: Number of animals and humans sampled in the 10 villages

Species	Number of animals and humans in each village: Number among included households/number sampled										
	Kibuye	Masolya	Makoma	Busakira	Busano	Nagongera	Isakabisolo	Busindha	Namungodi	Matyama	Total (%)
**Pig**	156/154	64/63	63/63	39/39	10/10	42/37	17/17	66/64	39/39	28/28	524/514 (98.1)
**Dog**	53/50	44/43	14/13	29/26	36/35	30/28	23/22	28/26	30/30	12/9	299/282 (94.3)
**Cat**	4/3	4/4	3/3	3/3	2/2	1/1	0/0	4/3	1/1	2/2	24/22 (91.7)
**Goats**	108/108	162/160[a]	222/222[b]	25/25	55/45	46/43	56/56	55/55	75/75	44/44	848/833 (98.2)
**Cattle**	26/26	31/22	18/12	7/4	6/4	27/14	13/13	5/5	3/3	12/9	148/112 (75.7)
**Rabbits**	4/4	-	2/2	-	-	-	-	-	-	-	6/6 (100)
**Sheep**	-	1/1	7/7	-	-	-	-	-	-	-	8/8 (100)
**Chicken**	430/64	286/42	388/55	140/45	224/44	178/49	333/45	246/42	217/43	141/38	2583/467 (18.2)
**Ducks**	69/28	10/6	29/12	26/10	22/10	44/16	22/7	8/3	12/5	7/3	249/100 (40.2)
**Pigeons**	45/3	4/1	45/7	-	-	12/3	4/2	1/0	27/5	2/0	140/17 (12.1)
**Turkeys**	7/1	-	10/1	-	-	-	-	5/2	-	-	22/4 (18.2)
**Guinea fowls**	-	-	-	-	-	-	-	6/1	4/2	4/0	14/3 (21.4)
**Rats[b]**	15	11	16	13	-	-	-	-	-	10	65
**Humans**	354/337	242/228	231/214	174/164	153/144	186/154	155/128	105/99	204/190	127/108	1931/1766 (91.5)

[a]Number includes goats (101 and 162 respectively) from additional households sampled on an additional criterion of having at least one goat; see materials and methods
[b]Number of rats trapped in the cages in various villages
