# Supplementary material for: Animal Reservoirs of Zoonotic Tungiasis in Endemic Rural Villages of Uganda
Source: PLoS Negl Trop Dis. 2015 Oct 16;9(10):e0004126. doi: 10.1371/journal.pntd.0004126 (PMC4608570; doi:10.1371/journal.pntd.0004126)
Supplement: S3 Table — This table illustrates the detailed bivariate analysis of factors of tungiasis in animals at household level (herds). (PDF) [file pntd.0004126.s004.pdf]

S3 Table. Animal tungiasis risk factors at household level

Factor	Sampled (n)	Affected (%)	OR (95% CI)	p-value
**Village**				
**Busindha**	14	10 (71.4)	95 (9.5-946.9)	0.000
**Masolya**	32	18 (56.3)	48.9 (6-400.1)	0.000
**Busakira**	19	6 (31.6)	17.5 (1.9-159.7)	0.01
**Makoma 1**	30	8 (26.7)	13.8 (1.6-117.9)	0.02
**Kibuye**	45	11 (24.4)	12.3 (1.5-100)	0.02
**Matyama**	15	3 (20)	9.5 (0.9-100)	0.06
**Namungodi**	25	3 (12)	5.2 (0.5-52.9)	0.17
**Busano**	17	2 (11.8)	5.1 (0.4-60.1)	0.2
**Isakabisolo**	20	1 (5)	Reference	
**Nagongera**	19	0 (0)	Excluded	
**Sex of household head**				
**Male**	192	53 (27.6)	1.5 (0.67-3.3)	0.33 (exact 0.45)
**Female**	44	9 (20.5)		Reference
**Ethnic group of household head**				
**Bantu**	85	20 (23.5)	1 (0.5-2.1)	0.9
**Nilo hamites (Itesot)**	51	19 (37.3)	2 (1-4)	0.07
**Luo (Japadhola)**	100	23 (23)		Reference
**Education level of household head**				
**None**	78	22 (28.2)	1.2 (0.4-3.2)	0.74
**Primary**	130	33 (25.4)	1 (0.4-2.6)	0.97
**Above primary**	28	7 (25.0)	Reference	
**Age of household head (Years)**				
**15-35**	51	18 (35.3)	2.1 (1-4.4)	0.05
**36-55**	111	23 (20.7)	Reference	
**56-87**	74	21 (28.4)	1.5 (0.77-3)	0.23
**Main source of income**				
**Subsistence agriculture**	220	58 (26.4)	0.9 (0.29-3.0)	0.9
**others**	16	4 (25.0)	Reference	
**Household size (number of people)**				
**1-5**	48	6 (12.5)	Reference	
**6-10**	138	41 (29.7)	2.96 (1.2-7.5)	0.02
**11-24**	50	15 (30)	3 (1.1-8.6)	0.04
**Homestead size**				
**1-3 households**	215	55 (25.6)	Reference	
**4-8**	21	7 (33.3)	1.5 (0.6-3.8)	0.44
**Household monthly income (USHS)**				
**5000-130000**	166	43 (25.9)	Reference	
**Above 130000**	70	19 (27.1)	1.1 (0.6-2.0)	0.84
**Human infestation**				
**Yes**	80	40 (50)	6.1 (3.3-11.4)	0.0001
**No**	156	22 (14.1)	Reference	

S3 Table continued

Factor	Sampled (n)	Affected (%)	OR (95% CI)	p-value
**Period human tungiasis was last experienced (months)**				
**0-3**	113	45 (39.8)	4.6 (2.4-9.1)	0.0001
**Above 3 (4-360)**	112	14 (12.5)	Reference	
**Never**	11	3 (27.3)	2.6 (0.6-11.1)	0.19
**Number of affected humans**				
**0**	156	22 (14.1)	Reference	
**1-4**	62	28 (45.2)	5.0 (2.6-9.8)	0.0001
**5-12**	18	12 (66.7)	12.2 (4.1-35.8)	0.0001
**Know cause**				
**Yes**	211	56 (26.5)	0.9 (0.3-2.3)	0.8
**Know**	25	6 (24.0)	Reference	
**Number of animal species in a household**				
**1-4**	207	50 (24.2)	Reference	
**5-9**	29	12 (41.4)	2.2 (1-5)	0.05
**Have pigs**				
**Yes**	155	55 (35.5)	5.8 (2.5-13.5)	0.0001
**No**	81	7 (8.6)	Reference	
**Number of pigs in a household**				
**0**	81	7 (8.6)	Reference	
**1-5**	133	46 (34.6)	5.6 (2.4-13.1)	0.0001
**Above 5**	22	9 (40.9)	7.3 (2.3-23.1)	0.001
**Have dogs**				
**Yes**	120	31 (25.8)	Reference	
**No**	116	31 (26.7)	1 (0.6-1.9)	0.9
**Have cattle**				
**Yes**	47	16 (34.0)	1.6 (0.8-3.2)	0.2
**No**	189	46 (24.3)	Reference	
**Cat present**				
**Yes**	19	6 (31.6)	1.3 (0.5-3.7)	0.55
**No**	217	56 (25.8)	Reference	
**Chicken present**				
**Yes**	203	51(25.1)	Reference	
**No**	33	11 (33.3)	1.5 (0.7-3.3)	0.3
**Other poultry**				
**Yes**	70	22 (31.4)	1.4 (0.8-2.7)	0.2
**No**	166	40 (24.1)	Reference	
**Animal rearing experience (years)**				
**1-5**	64	17 (26.6)	1.1 (0.5-2.2)	0.8
**6-10**	67	19 (28.4)	1.2 (0.6-2.4)	0.6
**Above 10**	105	26 (24.8)	Reference	
**Manure disposal distance from compound (meters)**				
**1-4**	135	40 (29.6)	1.8 (0.6-5.0)	0.3
**5-10**	75	17 (22.7)	1.2 (0.4-3.8)	0.7
**Above 10**	26	5 (19.2)	Reference	
**Method of manure disposal**				
**Pit/burn**	79	21 (26.6)	1.0 (0.6-1.9)	0.9
**Garden/bush**	157	41 (26.1)	Reference	
