# Supplementary material for: Animal Reservoirs of Zoonotic Tungiasis in Endemic Rural Villages of Uganda
Source: PLoS Negl Trop Dis. 2015 Oct 16;9(10):e0004126. doi: 10.1371/journal.pntd.0004126 (PMC4608570; doi:10.1371/journal.pntd.0004126)
Supplement: S4 Table — (PDF) [file pntd.0004126.s005.pdf]

S4 Table. Pig tungiasis herd risk factor analysis.

Factor	Sampled (n)	Affected (%)	OR (95% CI)	p-value
**Human infestation**				
**Yes**	56	35 (62.5)	7.0 (3.36-14.66)	0.0001
**No**	99	19 (19.2)	Reference	
**Number of pigs**				
**1-3**	113	34 (30.1)	Reference	
**4-6**	24	14 (58.3)	3.25 (1.32-8.045)	0.011
**7-20**	18	6 (33.3)	1.16 (0.403-3.35)	0.781
**Other pig parasites**				
**Yes**	138	53 (38.4)	9.98 (1.29-77.43)	0.028
**No**	17	1(5.5)	Reference	
**Pig management system**				
**Intensive**	1	0 (0)		
**Extensive**	154	54 (35.1)		
**Pig ectoparasites control**				
**Yes**	20	6 (30)	Reference	
**No**	135	48 (35.6)	1.29 (0.465-3.57)	0.627
**Pig ectoparasiticide used**				
**Pyrethroid**	6	3(50)	1.94 (0.378-9.96)	0.427
**Amitraz**	2	1(50)	1.94 (0.12-31.67)	0.642
**Don't know/traditional**	12	2(16.7)	Reference	
**Interval of pig ectoparasite control (weeks)**				
**1-4**	8	1 (12.5)	Reference	
**5-12**	12	5(41.7)	1.37 (0.41-4.54)	0.606
**System of drug application**				
**Wash**	7	4(57.1)	2.61 (0.56-12.13)	0.220
**Spraying**	13	2(15.4)	Reference	
**Pig housing**				
**Yes**	11	2 (18.2)	Reference	
**No**	144	52 (36.1)	2.54 (0.53-12.22	0.244
**Pig residence floor**				
**Concrete**	1	0(0)		
**Non concrete**	154	54(35.1)		
**Distance of pig confinement area from human compound (meters)**				
**0-10**	81	27(33.3)	1.75 (0.63-4.84)	0.281
**11-20**	47	21(44.7)	2.83 (0.97-8.28)	0.058
**>20**	27	6(22.2)	Reference	
**Dogs present**				
**Yes**	49	23 (46.9)	2.14 (1.06-4.31)	0.033
**No**	106	31(29.2)	Reference	
**Goats present**				
**Yes**	94	36 (38.3)	1.48 (0.74-2.96)	0.263
**No**	61	18 (29.5)	Reference	

S4 Table continued

Factor	Sampled (n)	Affected (%)	OR (95% CI)	p-value
**Chicken present**				
**Yes**	133	45 (33.8)	Reference	
**No**	22	9 (40.9)	0.74 (0.29-1.86)	0.520
**Cat present**				
**Yes**	13	5 (38.5)	1.19 (0.37-3.82)	0.78
**No**	142	49 (34.5)	Reference	
**Other poultry**				
**Yes**	45	19 (42.2)	1.56 (0.77-3.2)	0.219
**No**	110	35 (31.8)	Reference	
**Pig dwelling sanitation**				
**Manure soiled (Dirty)**	127	49(38.6)	2.89 (1.03-8.1)	0.044
**Clean**	28	5(17.9)	Reference	
**Dog tungiasis**				
**Yes**	9	7 (77.8)	7.4 (1.5-36.9)	0.015
**No**	40	16 (40)	Reference	
**Cattle present**				
**Yes**	29	13 (44.8)	0.6 (0.3-1.5)	0.21
**No**	126	41 (32.5)		
