# Supplementary material for: Animal Reservoirs of Zoonotic Tungiasis in Endemic Rural Villages of Uganda
Source: PLoS Negl Trop Dis. 2015 Oct 16;9(10):e0004126. doi: 10.1371/journal.pntd.0004126 (PMC4608570; doi:10.1371/journal.pntd.0004126)
Supplement: S5 Table — (PDF) [file pntd.0004126.s006.pdf]

S5 Table. Dog tungiasis risk factor analysis

Factor	Sampled (n)	Affected (%)	OR (95% CI)	p-value
**Human tungiasis**				
**Yes**	47	12 (25.5)	12.17 (2.58-57.39)	0.002
**No**	73	2 (2.7)	Reference	
**Dog number**				
**1-4**	103	11 (10.7)	Reference	
**5-10**	17	3 (17.7)	1.7 (0.44-7.23)	0.412
**Other parasites**				
**Yes**	116	14 (12.1)		
**No**	4	0 (0)		
**Pigs on the premises**				
**Yes**	49	8 (16.3)	2.11 (0.68-6.53)	0.194
**No**	71	6 (8.5)	Reference	
**Ectoparasites control**				
**Yes**	6	2 (33.3)	4.25 (0.7-25.7)	0.115
**No**	114	12 (10.5)	Reference	
**Goats on premises**				
**Yes**	84	11 (13.1)	1.66 (0.43-6.34)	0.46
**No**	36	3 (8.3)	Reference	
**Cattle on premises**				
**Yes**	29	6 (20.7)	2.7 (0.85-8.58)	0.091
**No**	91	8 (8.8)	Reference	
**Chicken on the premises**				
**Yes**	98	12 (12.3)	1.4 (0.29-6.73)	0.678
**No**	22	2 (9.1)	Reference	
**Cats on premises**				
**Yes**	9	3(33.3)	4.6 (0.99-20.77)	0.051
**No**	111	11 (9.9)	Reference	
**Other poultry**				
**Yes**	38	4 (10.5)	Reference	
**No**	82	10 (12.2)	1.18 (0.35-4.03)	0.791
**Pig infections**				
**Yes**	23	7 (30.4)	5.6 (1.7-18.2)	0.004
**No**	64	2 (3.1)	Reference	
